# Humpback whale adult females and calves balance acoustic contact with vocal crypsis during periods of increased separation

**DOI:** 10.1002/ece3.8604

**Published:** 2022-02-09

**Authors:** Katherine L. Indeck, Michael J. Noad, Rebecca A. Dunlop

**Affiliations:** ^1^ Cetacean Ecology and Acoustics Laboratories School of Veterinary Science University of Queensland Gatton Queensland Australia; ^2^ Present address: University of New Brunswick Saint John New Brunswick Canada

**Keywords:** acoustic crypsis, active space, behavioral states, communication strategy, contact calling, humpback whales, parent–offspring interaction, separation

## Abstract

Acoustic communication is important for animals with dependent young, particularly when they are spatially separated. Maternal humpback whales (*Megaptera novaeangliae*) use acoustic calling to help minimize the risk of separation from their young calves during migration. These pairs also use acoustic crypsis to minimize detection by males. How they balance a restricted active space with the need to maintain acoustic contact during periods of separation is not yet understood. Here, we analyzed movement metrics of tagged adult female–calf pairs during migration to identify two behavioral states, “resting/milling” and “travelling.” When travelling, these pairs dived synchronously and exhibited little to no spatial separation. Alternatively, adult females had significantly longer dive durations (*p* < .01) when resting, and while they spent prolonged times at depth, calves would surface several times independently. This demonstrated that these pairs are frequently separated during periods of rest. We then determined whether the call rates and acoustic levels of these pairs increased with more frequent separation, finding that both adult females and calves significantly increased their call rates, but not levels, when resting. We also found that adult female–calf pairs have a restricted active space, with less than 15% of calls estimated to be detectable beyond 2 km. However, as with call level, detection distance did not differ significantly between the two behavioral states. In summary, adult female–calf pairs maintain successful communication during periods of separation by calling more frequently rather than by producing louder calls. This strategy aids in maintaining acoustic contact while simultaneously limiting detectability by conspecifics.

## Graphical abstract

Maternal humpback whales (*Megaptera novaeangliae*) must balance acoustic calling to help minimize the risk of separation from their young calves during migration, with acoustic crypsis to minimize detection by males. We found that adult female–calf pairs maintain successful communication during periods of separation by calling more frequently rather than by producing louder calls. This strategy aids in maintaining acoustic contact while simultaneously limiting detectability by conspecifics.

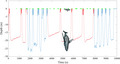

## 1 INTRODUCTION

Parent–offspring contact calls are especially important for animals with dependent young (Rendall et al., 2000; Sousa‐Lima et al., 2008; Trimble & Insley, 2010). As such, adults must develop a means of efficiently maintaining contact with their offspring. This is particularly important when the risk of spatial separation increases because, if separated, young animals are highly susceptible to predation or starvation (Rendall et al., 2000). However, the likelihood of separation is reduced during certain behavioral states due to the evolution of an “innate following tendency” in young animals (Rendall et al., 2000; Thomas & Taber, 1984). For example, as rates of travel increase, offspring will inherently stay close to their parent. However, when separation does occur, preventing visual and physical contact, communication can be maintained by using contact calls. These calls are used to coordinate both close‐ and long‐range exchanges between conspecifics across terrestrial and marine taxa and often contain socially relevant information, including the sender's identity, providing a means of facilitating reunions and enabling individual recognition (Arnold & Wilkinson, 2011; Kondo & Watanabe, 2009; Sousa‐Lima et al., 2002). Therefore, they are frequently emitted during periods of parent–offspring separation, as noted in marine species such as bottlenose dolphins (*Tursiops* sp.), manatees (*Trichechus* sp.), and South American sea lions (*Otaria flavescens*; Smolker et al., 1993; Sousa‐Lima et al., 2002, 2008; Trimble & Insley, 2010).

The efficacy of contact calling depends upon the ability of signals to reach their intended receiver. The detection limit of any given call (i.e., the spatial extent to which it is audible to the target individual) influences the communication active space (Brenowitz, 1982; Clark et al., 2009; Janik, 2005). Under natural noise conditions, “active space” is determined by: (1) the level of the signal; (2) the signal's temporal and spectral acoustic features; (3) the propagation properties of the environmental medium; (4) the level of ambient noise; and (5) the detection threshold of the intended receiver, which is the lowest limit that a signal is audible when embedded in the background noise (Brenowitz, 1982). As a result, active space can be spatially extensive and temporally variable, meaning that parent–offspring calls that are directed toward one another may also be detected by unintended bystanders (e.g., predators and/or conspecifics; Janik, 2005; Peake, 2005). For maternal females, this can lead to unwanted, agonistic interactions, particularly with males of the same species, that can pose a direct physical threat to the female herself, as well as endanger her offspring's health and development (Boness et al., 1995; Chilvers et al., 2005; Sundaresan et al., 2007; Weir et al., 2010). Therefore, the active space of parent–offspring contact calls must be large enough to include each other but small enough to reduce the probability of including nearby conspecifics. As such, it would be reasonable to assume that parent–offspring active space is designed to be dynamically cryptic to maximize contact while minimizing detection. Consequently, if spatial separation increases between a parent and its offspring, so too might the active space of their calls, despite increased risk of being overheard.

Cetaceans (whales, dolphins, and porpoises) are an acoustically active group of marine mammals that invest a great deal of energy in the development of their calves (Rendell et al., 2019). Separation, however, is an ever‐present risk for highly mobile species in the vast marine environment as visibility is usually poor (tens of meters, often equating to only a few body lengths; e.g., Eiras et al., 2021). Fortunately, sound is transmitted very efficiently in water, making vocal signals a highly useful way of maintaining contact with precocial young until they reach independence. For example, postpartum use of individualized signature whistles in bottlenose dolphins is important in facilitating mother–calf reunions (King et al., 2016), while family‐specific call types in killer whales, *Orcinus orca*, are thought to enhance the ability of new calves to recognize family members and maintain contact with the group (Weiß et al., 2006). Likewise, sperm whales, *Physeter macrocephalus*, have developed distinctive mother–calf coda repertoires that are significantly different to those used by most other unit members, which is thought to aid mother–calf interaction following separation (Schulz et al., 2011). It is also likely that other aspects of cetacean calling behavior (e.g., call rate and level) are similarly influenced by behavioral activities with an increased potential of adult female–calf separation.

The seasonal migrations of humpback whales (*Megaptera novaeangliae*), between tropical breeding grounds and polar foraging areas, are challenging for postcalving females as they must remain in contact with their young calves while travelling long distances in a low visibility environment. Therefore, contact calling is likely fundamental for calf survival. Humpback whale adult female–calf calls are commonly heard on the breeding grounds and during migration (Dunlop et al., 2008; Videsen et al., 2017; Zoidis et al., 2008). They exhibit considerable differences in their acoustic parameters between age classes (Indeck et al., 2021) and the variety of call types produced suggests that they may serve several potential functions, including as contact, distress, and/or nursing calls. Additionally, Videsen et al. (2017) noted that adult female–calf pairs produced calls at very low levels (on average 136 and 141 dB re 1 µPa, depending on call type) compared to previously reported calls by other humpback whale groups (e.g., Dunlop et al., 2013) and suggested that these resulted in a restricted active space with a radius of only 30 m. Cryptic calling, resulting in calls that are naturally difficult to detect, has been shown to be a vocal strategy for avoiding unwanted conspecific interaction (Indeck et al., 2021). However, such low‐level calls would be particularly susceptible to acoustic masking from anthropogenic activities, increasing the risk that a female may lose acoustic contact with her calf.

Although previous estimates indicate that the adult–female calf active space is quite limited, it is still unknown how these pairs balance acoustic crypsis with the need to maintain contact, particularly when separated. While migration is predominantly characterized by travel, on a fine scale, humpback whale groups exhibit a variety of behavioral states along these routes (Kavanagh, 2014). Behavioral states are characterized by differences in movement patterns (such as speed, course, and diving/surfacing metrics), which lead to varying degrees of separation risk for adult females and their calves. Therefore, we quantitatively defined the behavioral states of these pairs on their migratory route along eastern Australia and determined which one is most representative of potential separation. We then investigated if/how they modified their calling activity in response to their risk of separation (assuming that as the risk increased, so too would their call rates and/or levels). Further, we established a robust estimate of the adult female–calf active space by using a site‐specific propagation model and determined whether active space changed in response to more frequent spatial distancing within these pairs. This provided the contextual framework for examining how adult female–calf humpback whales successfully balance acoustic crypsis, where they vocalize quietly and infrequently to avoid eavesdroppers (Indeck et al., 2021), with the need to maintain contact during periods of separation.

## 2 MATERIALS AND METHODS

### 2.1 Data collection

Data were collected in September and October, during the whales’ southward migration away from the breeding grounds, across 4 years off the coast of Peregian Beach, Queensland, Australia (26°29′S, 153°06′E). We attached high‐resolution digital acoustic tags (DTAGs) onto adult females travelling with a calf in 2010, 2011, and 2014. In 2017, two Acousonde acoustic tags (Greenridge Sciences, Inc.) were deployed simultaneously, one on the adult female and one on her calf. Both types of tags are lightweight, noninvasive, temporary attachments held in place by suction cups. They contain a hydrophone and multiple accelerometers, magnetometers, and a pressure sensor providing data on orientation, underwater movement, and depth (Johnson & Tyack, 2003). The DTAGs recorded 16 bit audio at a sampling rate of 48 or 96 kHz, depending on the year; no acoustic data were used from the simultaneously deployed Acousondes (see below). No repeated tagging of individuals occurred, as confirmed through photograph identification. Please note that adult females in this study were presumed to be the mothers of the calves they were with, but due to observations on several occasions that challenged the accuracy of this assumption, we opted for the term “adult female.”

Detailed data collection methodology has been presented elsewhere (e.g., Dunlop et al., 2015, 2017, 2020) and is summarized here. In 2010, 2011, and 2014, detailed behavioral data were collected from land‐ and boat‐based focal follows for the duration of the DTAG deployments. Once a tag was deployed, the tagging vessel followed each focal pair of whales at a distance of 100–200 m, which allowed for the collection of detailed behavioral observations of each individual while minimizing disturbance to the adult female and her calf. Simultaneous land‐based visual observations were conducted from two elevated survey points to the north (“North Station”) and south (“Emu Mountain Station”) of Peregian Beach. A surveyor's theodolite connected to a laptop running the computer software VADAR (Visual and Acoustic Detection and Ranging; Eric Kniest, University of Newcastle) allowed each focal group of humpback whales to be continuously tracked in real time for up to 7 h and out to approximately 15 km from shore, as they passed through the survey area. Focal follow observations from both land and vessel platforms were used to build a comprehensive record of each tagged group's composition, social affiliations, surface behaviors, and movement for the duration of the tag deployment. To ensure a standard group composition across the study, only tags from adult female–calf pairs that did not interact with (i.e., join and form a group with) any other whales over the course of tagging/focal follow were analyzed (*n* = 15).

In 2017, when both an adult female and her calf were tagged, the calf was tagged first. An hour later, we tagged the adult female. After tagging, we followed the pair at a distance of approximately 300 m, recording changes in group composition and visible energetic surface behaviors. As the double‐tagged pair was escorted by other whales for nearly the entirety of the ensuing focal follow, no acoustic or fine‐scale movement data from these tags were used for analyses, as our focus here was on unaccompanied pair behavior. As such, the 2017 tags are included exclusively to demonstrate the simultaneous dive behavior of the female and the calf as a means of validating our assumptions of separation risk between behavioral states.

### 2.2 Identifying adult female–calf behavioral states

The movement of tagged females was measured continuously. This time series was broken into 10‐min bins beginning 10 min after tag deployment, or at the start of the corresponding focal follow (if initiated more than 10 min after tagging). This time offset was used to minimize the inclusion of temporary behavioral or vocal changes in response to tagging; this is sufficient time for pairs to return to much of their “pre‐tagging” behavior (Williamson et al., 2016). Using the dual land‐ and boat‐based focal follow data (which recorded surfacing by one or both individuals every 4–7 min, on average), the positions of a group at the start and end of each time bin were interpolated, assuming the whales maintained a straight line and constant speed of travel from the last recorded position in one bin to the first recorded position in the following bin. These interpolated positions were then used to estimate swimming speed (km/h) and course travelled (degrees) for each bin.

In addition to swimming speed, “speed south” (km/h), a measure of migratory movement, was also calculated by considering only southerly displacement, or the whales’ latitudinal movement, during each time bin. For example, a negative speed south indicated a net northward movement of the whales over the 10‐min bin. Alternatively, a positive value represented southerly travel parallel to the coast, and a value close to zero indicated either stationary behavior or east/west movement toward or away from shore. In addition to compass course, “course deviation” (degrees) was also determined as the absolute value of the difference in course from the previous to the current bin, giving a maximum value of 180° (i.e., swimming in the opposite direction). Low course deviation values from one time bin to the next (e.g., those close to zero) suggested that whales were travelling in a relatively straight direction, while higher values (e.g., those >45°) were considered to be the result of milling behavior. Finally, the number of energetic surface behaviors per pair (e.g., breaches, pectoral slaps, fluke flaps) was counted for each 10‐min time bin.

Pressure sensor data from the DTAGs were used to divide the whales’ diving behavior into deep dives and surface intervals. Deep diving intervals were characterized by dives greater than 10 m in depth and longer than 75 s, to eliminate shallow dives attributable to incidental body movements near the surface (Dunlop et al., 2015; Kavanagh, 2014; Stimpert et al., 2012). Alternatively, surface intervals comprised of a series of successive shallow dives (<10 m) with several repeated surfacing to breathe. We used this to calculate the proportion of time spent at the surface (per 10 min), as well as the number of deep dives initiated per bin (as per the tagged adult female). The pressure data from the simultaneously deployed Acousondes in 2017 were used to create depth profiles of both the adult female and the calf, which were subsequently superimposed for a comparison of individual dive behavior.

The six variables calculated above (i.e., swimming speed, speed of southerly travel, course deviation, the number of energetic surface behaviors per pair, the proportion of time spent on the surface, and the number of deep dives) were then used to define the behavioral states of adult female–calf pairs during migration, resulting in a behavioral state for every 10‐min period. A total of 13 focal follows were included in this analysis, ranging in length from two to 23 time bins.

Behavioral states were defined using the nonhierarchical partitioning clustering method “k‐means” from the *stats* package in R (R Development Core Team, 2019). Each data point represented one 10‐min time bin that included calculations of the six variables chosen to characterize humpback whale migratory behavior. Prior to analyses, all variables were standardized to unit variance (i.e., converting the original measurements into unitless values) to account for differing measurement units across variables (e.g., km/h and degrees). Any time bins with missing movement metrics (e.g., the first time bin of each tag for which course deviation could not be calculated) were excluded from analyses. We then used the *NbClust* package in R (Charrad et al., 2014) to quantitatively determine the optimal number of clusters to be extracted for the *k*‐means method, which requires this to be specified before completing the analysis. A *k*‐means analysis was then conducted, separating time bins into the objectively predetermined number of clusters in such a way that minimized and maximized within‐ and between‐cluster variation, respectively. The nonstandardized means of the movement variables were calculated for each cluster and behavioral states proposed based on these values. These analyses resulted in two distinct clusters, so two behavioral states were identified for the tagged pairs in this study, which were used in subsequent analyses.

As calves were often observed on the surface while adult females were on a prolonged dive, depth was considered a proxy for potential risk of separation. Therefore, to examine whether fine‐scale dive parameters of the adult females were significantly different between behavioral states, we ran binomial generalized linear mixed‐effects models (LMM) using the *glmer* function from the *lme4* package in R (Bates et al., 2015). Behavioral state (as determined above) was the binary response variable, and the adult female's dive duration, mean dive depth, and maximum dive depth were the predictor variables. Tag ID was included in the models as a random effect. Models were ranked according to their AIC values and the model with the lowest value was selected as the one that best represented the relative importance of predictor variables. *p*‐values of <.05 were considered statistically significant. Observed means with 95% confidence intervals of significant variables are reported.

### 2.3 Vocal activity in response to separation risk

Each time bin was assigned a behavioral state based on the k‐means cluster analysis described above. After assignment, the number of calls during each time bin was determined by the visual and aural inspection of audio spectrograms created from the tag recordings, which enabled us to determine the call rate of each bin and, therefore, call rates for each behavioral state. The received levels (dB re 1 µPa) of these calls were measured using custom‐written MATLAB algorithms (Girola et al., 2019; The MathWorks Inc., 2017). Calls were provisionally assigned to the adult female or the calf, as per Indeck et al. (2021).

To determine whether adult female and/or calf call rates (calls per hour per age class) varied between behavioral states, generalized linear mixed models (GLMMs) were run using the *glmmTMB* package in R (Brooks et al., 2017). The GLMMs were run with a log offset of time (in decimal hours) and a negative binomial distribution with a log link and quadratic parameterization, to account for overdispersion, noninteger values, and zero‐inflated data. Group ID was included as a random effect to account for the lack of independence of calls recorded on the same tag. It is important to note that we present a minimum estimation of calf call rate, as adult female–calf separation distance likely resulted in some calf calls being missed by the tags on the adult females.

To determine whether adult females modified their received call level (dB re 1 µPa) in response to the behavioral state of the pair, a LMM was run using the *lmer* function from the *lme4* package in R (Bates et al., 2015). The LMM used Tag ID as a random effect, as well as a restricted maximum likelihood (REML) for unbiased estimates of variance components.

We analyzed only the received levels of those calls presumed to have been produced by the tagged adult female, as these were considered a proxy for source level and could most accurately be tested for changes in response to behavioral state. As tags were placed similarly on each adult female (i.e., a similar distance from the source, which is presumed to be the larynx), changes in source level should be reflected in changes in measured received level (Parks et al., 2019; Videsen et al., 2017). Alternatively, as we had no fine‐scale measure of calf distance from the adult female (and the tag) contemporaneous to each call recorded on the singly deployed DTAGs, we opted against testing received levels of presumed calf calls as it would have been impossible to determine if any resulting changes were due merely to calf separation distance or an actual response to behavioral state.

The *emmeans* package (Lenth, 2020) was then used post hoc to provide least squares means, which are adjusted to predict the effect of the factor variables on the response assuming equal sample sizes and, therefore, are more robust for unbalanced data than are observed averages (Harvey, 1960). Pairwise comparisons of age class call rates and adult female received levels between the two behavioral states were calculated using the “multivariate t” adjustment method, as it takes into consideration the correlation structure of the model (Lenth, 2020). Within‐model results are presented as t‐values and associated *p* values, with significance set to *p* < .05.

### 2.4 Adult female–calf active space estimation

To determine the communication active space of adult female–calf vocal signals (including calf calls), we estimated the potential detection distance for each call, based upon the received level, peak frequency, and corresponding wind‐dominated broadband noise level, following the methodology developed by Dunlop (2018b). Calls were only included in this analysis if ambient noise could be measured during the period of call production without audible boats or nearby singing whales (i.e., within 10 km). In 2010, 2011, and 2014, this noise was measured using an array of five hydrophone buoy systems, which was configured in a “T” shape and deployed in 18–25 m of water, approximately 1.5–2.5 km offshore of Peregian Beach (Dunlop et al., 2015; Noad et al., 2004). Array acoustic recordings included the times of all tag deployments and associated land‐based observations, meaning that wind‐dominated broadband noise levels could be determined for nearly every call recorded by each tagged adult female–calf pair.

To estimate these noise levels, a 10‐s noise sample was used every 10 min beginning at the first recorded call of the tagged adult female–calf pair. For each sample, broadband noise was measured over the 40 Hz to 2.5 kHz 1/3 octave bands using MATLAB. This bandwidth was chosen because almost all the energy contained within adult female–calf calls lies within this frequency range. Additionally, this band encompassed the peak frequencies of wind‐dominated noise at this study site. There was no evidence that system electronic noise contributed to our measures of background noise. As wind speed was constant over the study area when measurements were made, it is reasonable to assume that the broadband noise level recorded on the array was similar to that at the tagged adult female–calf pair.

Although the detection limit of humpback whale hearing is currently unknown, the audible threshold of a call was assumed to be when the signal‐to‐noise ratio (SNR) was equal to zero (following Dunlop, 2018b). The SNR was estimated as the difference between the measured call received level and the background noise level. How far each call was estimated to propagate before the SNR = 0 was then calculated using a site‐specific frequency‐ and distance‐dependent transmission loss equation. Depth‐constant (10 m) transmission loss was measured at this study site by playing octave band‐limited white noise from a source boat that ran transects toward and away from the hydrophone array, as described in detail in Dunlop et al. (2013). This resulted in the following regression equation:

TL=a+blog(x),

where *a* is a frequency‐dependent constant, *b* is the slope of the regression line, and *x* is the distance (in meters) from the source. While *b* varied with distance for most frequencies, two values (one applying to distances less than, and one to distances greater than, a crossover point where the slope changed) could be used to approximate it (Appendix, Table A1). Transmission loss values of each call were calculated depending upon the octave center frequency band that contained the call's peak frequency. However, it is important to note that specific differences in transmission loss due to depth have not been measured at this study site yet, so we were unable to account for caller depth and any resulting effects this may have on call detection distance. This is believed to have resulted in an overestimation of detection distances for calls produced near the surface. Alternatively, by including the received levels of calf calls (not just the apparent source levels of adult female calls), some detection distances are thought to have been underestimated.

We then used a generalized additive model framework, as per Dunlop (2018a, 2018b), to estimate the change in detection distance (response variable) as a function of call received level and broadband noise level (predictor variables). This was carried out using the packages *MRSea* and *geepack* in R. A complex region spatial smoother (CReSS) was used to fit a two‐dimensional smooth surface to the interaction between measured received level (*x*) and broadband wind‐dominated noise level (*y*). Using a spatially adaptive local smoothing algorithm (SALSA), the two‐dimensional surface was modeled according to the relationship between *x* and *y* (i.e., changes in measured received levels as a function of increasing background noise). A Bayesian information criteria were used to select the number and location of knots, which equated to flexibility in the surface resulting from differences in the *x* − *y* interaction. CReSS was then used to manipulate and smooth this flexibility. The resulting surface model represented an integrated measure of call received level and broadband noise level. This was then used as a covariate in the final model, which included detection distance as the response variable. This model was re‐run using a generalized estimating equation. Model predictions were used to create a figure that illustrates the integrated relationship between signal received level, broadband noise level, and the detection distances of each call.

Finally, to determine whether the detection distance of each adult female and calf call (response variable) differed significantly between behavioral state (predictor variable), a generalized LMM was run using Tag ID as a random effect and a gamma distribution with a log link (suitable for non‐normal data and/or values exclusively ≧0), using the *glmer* function from the *lme4* package (Bates et al., 2015).

## 3 RESULTS

### 3.1 Female–calf behavioral states

**FIGURE 1:**
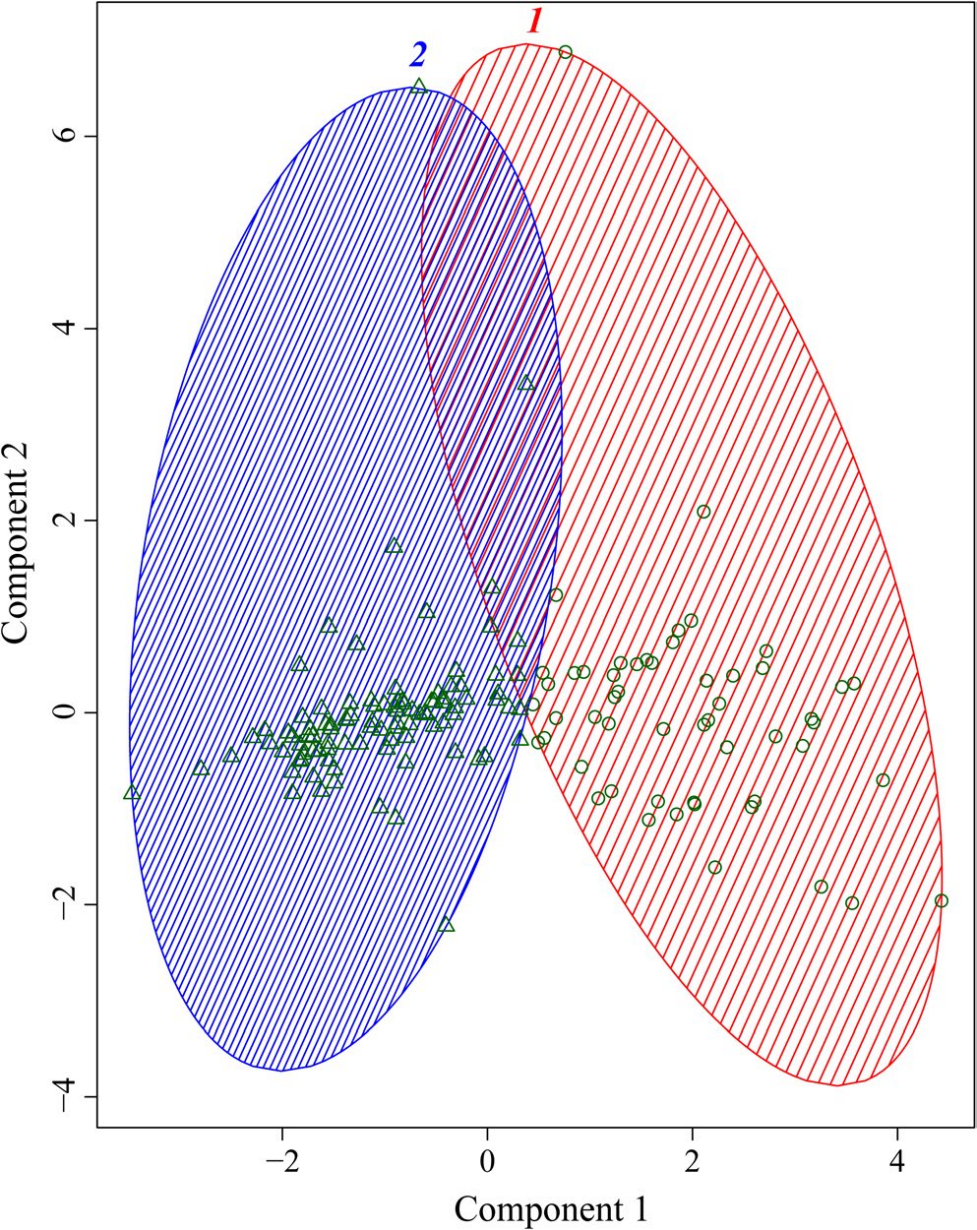
Cluster analysis illustrating cluster 1 (time bins in the resting/milling behavioral state, represented by circles) and cluster 2 (time bins in the travelling behavioral state, represented by triangles). The two components used to construct the clusters explain approximately 63% of the point variability. Component 1 is strongly influenced by speed and dive frequency (fast to slow from left to right along the *x*‐axis) and component 2 by energetic surface behaviors and proportion of time at the surface (high to low from bottom to top along the *y*‐axis)

Cluster 1 from the *k*‐means analysis was characterized by slow swimming speed, southward movement close to zero, and high course variation, whereas Cluster 2 was characterized by average migratory swimming speeds (Noad & Cato, 2007), low course variation, a lower proportion of time spent at the surface, and a higher frequency of deep dives (Table 1). Both clusters were characterized by very few energetic surface behaviors per group (<1 per 10‐min). Based on the means of the nonstandardized movement metrics for all time bins in each of the clusters (as presented in Table 1), whales in the time bins assigned to cluster 1 were considered to be in a “resting/milling” behavioral state, while those in cluster 2 were deemed to be in a “travelling” state (Figure 1; Jenner & Jenner, 2011; Kavanagh, 2014). Please note that these behavioral states are not all‐inclusive of those exhibited by adult female–calf pairs or other humpback whale groups during migration. They simply represent the behavioral states that were quantitatively determined given the movement characteristics of the 15 tagged pairs in this study.

**TABLE 1:** Nonstandardized mean values, with associated standard errors in brackets, of the movement metrics for each identified cluster

Cluster	1	2
Speed (km/h)	1.7 (±0.2)	4.6 (±0.1)
Speed south (km/h)	0.3 (±0.2)	4.1 (±0.1)
Course deviation (°)	91 (±11)	21 (±3)
No. ESB per group	0.30 (±0.16)	0.21 (±0.10)
Proportion of time at surface	0.34 (±0.04)	0.20 (±0.01)
Dive frequency	0.83 (±0.09)	1.79 (±0.07)
No. of time bins	53	97
**Proposed behavioral state**	**RESTING/MILLING**	**TRAVELLING**

Note

The number of time bins and proposed behavioral state for each cluster is also indicated. ESB = energetic surface behavior; dive frequency = number of deep dives initiated per 10‐min time bin.

**FIGURE 2:**
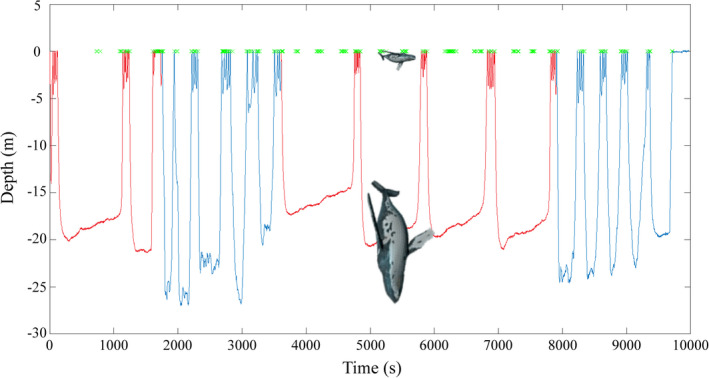
An example of an adult female dive profile demonstrating differences in dive duration between resting (in red; average = 14 m 42 s) and travelling (in blue; average = 4 m 45 s) behavioral states. The plot also includes focal follow observations of the calf at the surface (green *x*'s), illustrating increased separation during periods of resting. Interestingly, this tagged female was vertical in the water column, with her rostrum pointed downward, during her periods of rest, indicating that she was likely sleeping and/or facilitating the calf's nursing by positioning her mammary glands closer to the surface (whales are roughly to scale relative to water depth)

**FIGURE 3:**
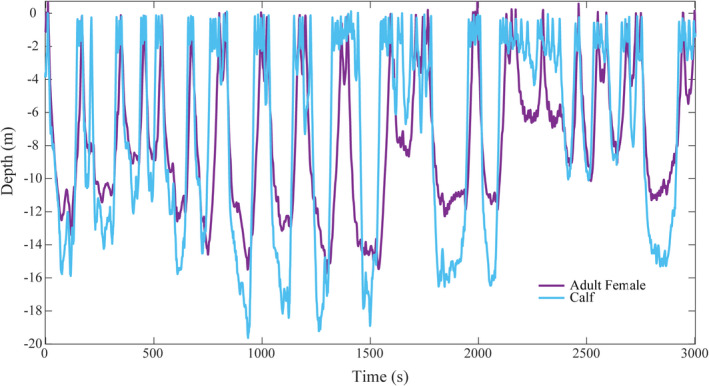
The depth profiles from the simultaneously deployed Acousondes, with the adult female profile in purple and the calf profile in light blue. These profiles occurred when the pair was travelling, demonstrating reduced dive durations during this behavioral state and minimal separation. Although it appears that the calf routinely dived deeper than the adult female, this was believed to be an artifact of tag position on the dorsum of the much higher mother. The calf was likely swimming in echelon around the level of the mother's ventrum

Duration was the only dive variable that differed significantly (*p* < .01) between resting (mean = 6 min 54 s; 95% CIs 05:35, 08:12) and travelling (mean = 4 min 22 s; 95% CIs 04:06, 04:39; Figures 2 and 3). Despite spending a greater proportion of time on the surface when resting, adult females and calves tended to separate more often during this behavioral state because the dives females did make were prolonged deep dives during which calves returned to the surface to breath multiple times (Figure 2). Alternatively, when travelling, these pairs were rarely separated, because although they dove more frequently and spent less overall time near the surface, adult female dive times were significantly shorter and calves synchronized their surfacing behavior to that of their mothers (Figure 3).

### 3.2 Vocal activity in response to separation risk

As predicted, the call rates of both adult females and calves were significantly higher when resting (4.6 calls/h) than while travelling (1.6 and 1.9 calls/h), as their separation risk was more frequent and at greater distances during these periods (Table 2). However, adult female received levels were not found to differ significantly between behavioral states (Tables 2 and 3), suggesting that these pairs chose to call more often, but not at louder levels, when separated.

**TABLE 2:** Results of the GLMMs comparing call rates (calls/hour/age class) and the LMM comparing adult female received call level (dB re 1 µPa), between behavioral states

Individual	Call rate (calls/h; ±SE)		Resting versus travelling		
	Resting	Travelling	Estimate (±SE)	*z*‐value	*p*‐value
Adult female	4.6 ± 1.7	1.6 ± 0.5	−1.07 ± 0.41	−2.59	**<.01**
Calf	4.6 ± 1.7	1.9 ± 0.5	−0.90 ± 0.43	−2.10	**<.05**

Individual	Received level (dB re 1 µPa; ±SE)		Resting versus travelling		
	Resting	Travelling	Estimate (±SE)	*t*‐value	*p*‐value
Adult female	147 ± 2.35	146 ± 2.57	−1.61 ± 3.0	−0.53	.63

Note

A negative emmeans estimate and z/t‐value indicates lower call rates and received level in the “travelling” behavioral state versus “resting.” Significant results are highlighted in bold.

**TABLE 3:** Results of the generalized linear mixed‐effects model comparing the combined detection distance (m) of adult female–calf calls (i.e., their active space) between behavioral states

Detection distance (m; ±SE)		Resting versus travelling		
Resting	Travelling	Estimate (±SE)	*t*‐value	*p*‐value
837 ± 184	565 ± 132	−0.39 ± 0.28	−1.42	.15

Note

A negative emmeans estimate and t‐value indicates a greater overall detection distance for the “resting” behavioral state versus “travelling.” However, the result was not significant at the *p* < .05 level.

**FIGURE 4:**
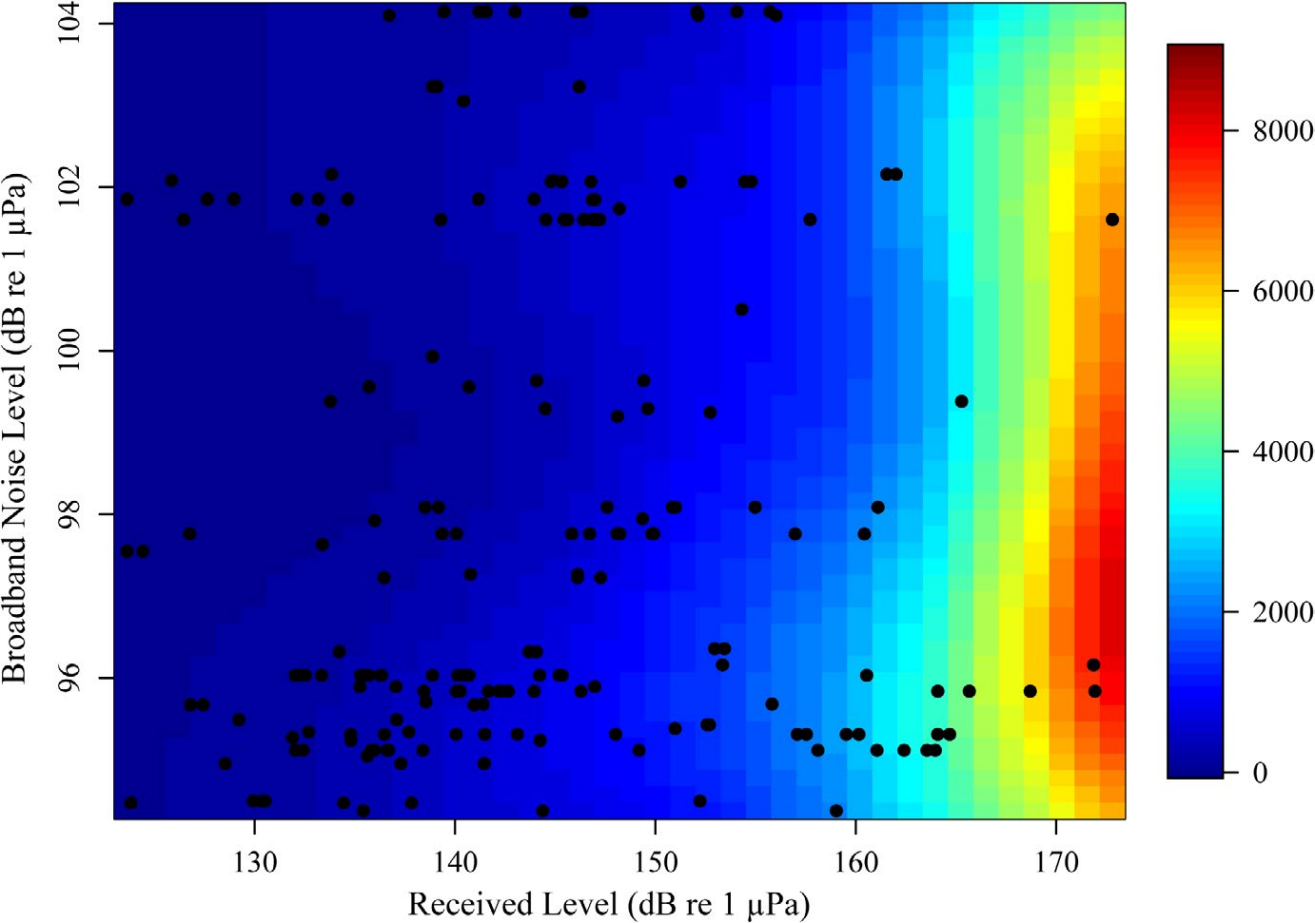
The relationship between the received level (dB re 1 µPa) of the signal (*x*‐axis), the wind‐dominated broadband noise level (dB re 1 µPa; *y*‐axis), and the estimated detection distance (m) of adult female–calf calls (color bar). Black dots represent each individual call

Received level of presumed adult female calls (i.e., a proxy for source level) ranged from 124 dB re 1 µPa to 172 dB re 1 µPa (median and mean ~148 dB re 1 µPa), which is comparable to social sound source levels measured from far‐field recordings by Dunlop et al. (2013). The range of received levels of presumed calf calls was 124 dB re 1 µPa to 173 dB re 1 µPa (median and mean ~140 dB re 1 µPa). Wind‐dominated broadband noise level ranged from 94 dB re 1 µPa to 104 dB re 1 µPa (median = 96 dB re 1 µPa; mean = 98 dB re 1 µPa), equating to wind speeds of approximately 8–13 knots, which were considered mid‐level for this study site. The final statistical model output for active space is presented below, with the measured response variable on the left and the significant predictor variable (the combined effect of received level and broadband noise level, with degrees of freedom) on the right. Figure 4 displays the significant relationship between the received and broadband noise levels (*x* and *y*) and detection distance.

Distance∼s(receivedlevel,broadbandnoiselevel,d.f.=10)

The estimated detection distance (to SNR = 0) for adult female–calf calls ranged between approximately 17 m and 8 km (median distance = 490 m; mean distance = 895 m), depending on the received level of the signal and the background broadband noise level (Figure 4). By a distance of 2 km, 86% of adult female–calf calls had reached an SNR of 0, the majority of which had a received level below 150 dB re 1 µPa. Of the remaining 14% with an estimated detection distance beyond 2 km, every call had a received level greater than 150 dB re 1 µPa.

Like received levels, the combined estimated detection distance (i.e., the adult female–calf active space) did not differ significantly between behavioral states. Nevertheless, it is interesting to note that while not statistically significant, the active space of these pairs was found to be nearly 300 m less when travelling than while resting (Table 3). This was due largely to the fact that the octave center frequency band that contained the peak frequencies of the most calls differed between states (mode = 500 Hz when travelling versus 125 Hz while resting), indicating that adult females and calves may use different call types or lower the peak frequency of the same call types to enhance their detection distance in response to increased risk of separation.

## 4 DISCUSSION

Maternal humpback whales invest heavily in the care of their calves, so the ability to maintain contact during a long migration is essential for calf survival. Acoustic communication in animals is context‐dependent; individuals often modify the acoustic features of their calls (e.g., call rate, duration, or frequency) when engaged in an activity that increases their risk of separation from certain others of their species (Rendall et al., 2000; Sugiura, 2007). Here, we found that this risk was greatest for adult female–calf humpback whale pairs during periods of “resting/milling” as compared to periods of “travelling.” As expected, these pairs called significantly more often when resting; however, they did not increase their call levels between behavioral states. Due to their consistently low levels, the majority of adult female–calf calls were estimated to reach an SNR of 0 before 2 km, limiting their detectability by nearby conspecifics (Indeck et al., 2021). Therefore, we show that these pairs balance their need to maintain contact when separated (i.e., increased calling) with their effort to simultaneously maintain acoustic crypsis (i.e., quiet calling).

During their southward migration, we identified two adult female–calf behavioral states, which represented very different degrees of separation risk. Though swimming speeds were greater when travelling, adult female–calf separation during these periods was infrequent and brief. Young offspring inherently stay in close proximity to their mothers as rate of travel increases due to an “innate following tendency” (Rendall et al., 2000; Thomas & Taber, 1984). Making shorter, more frequent dives during directed travel is thought to be a strategy of adult female humpback whales to maximize physical proximity to their calves. The simultaneous depth profiles of a maternal female and her calf when traveling (Figure 3) illustrates this nicely, demonstrating that these pairs tend to dive synchronously during these periods, with little to no prolonged separation. Alternatively, adult females were frequently separated from their calves during periods of rest. This was due to extended deep‐diving events that were significantly longer than dives performed while travelling and were interspersed with the calf surfacing multiple times independently. The long, slow rise and vertical body positioning exhibited by the adult female in Figure 2 indicates that during these periods, mothers may spend part of their time at depth sleeping, gradually drifting upward due to a slight positive buoyancy. Although adult females are not expected to call while asleep, the need to communicate with their calves directly following these periods would contribute to the rates of calling observed here. As such, acoustic contact during migration is arguably most vital during periods of rest when the calf is more spatially itinerant than the adult females and prone to increased horizontal and vertical separation, resulting in higher call rates from both individuals.

The levels of adult female–calf calls, however, remained consistently low regardless of behavioral state. While we did find that some adult female–calf calls were only estimated to propagate short distances (i.e., 19 were predicted to have an active space of less than 100 m), we also found the active space for the majority of adult female–calf calls to be well beyond the 30 m that Videsen et al. (2017) reported. There may be several reasons for this. Differences in analytical methods undoubtedly contributed to the difference in active space between studies, as we used a site‐specific frequency‐ and distance‐dependent transmission loss equation that included noise measurements temporally specific to individual calls and differentiated between noise contributors (e.g., natural vs. anthropogenic sources). Furthermore, their study focused on adult female–calf pairs on the calving grounds, where most calves are smaller and remain more consistently close to their mothers than those during migration. Indeed, they found that these pairs vocalized less frequently when resting than while on active dives (opposite to the results of our analyses), which is believed to be the result of differences in calf dependency and behavioral priorities on the calving grounds (nursing and rest) versus migration (travel).

Compared to other groups of humpback whales (e.g., those containing male escorts) from the same population and study site, however, these pairs do exhibit a restricted communication space. For example, Dunlop (2018b) estimated that the average active space of all humpback whale nonsong calls in low (< 95 dB re 1 µPa) to median (100 dB re 1 µPa) wind noise was 4 km. In contrast, we found that by a distance of 2 km, in similar wind noise, over 85% of adult female–calf calls were below an SNR of 0. Nevertheless, it would be reasonable to assume that acoustic crypsis would select for calls that are detectable at smaller distances than some of those estimated here. However, there is the occasional necessity for calls from these pairs to travel further than intuitively expected, as separation distances between adult females and calves have the potential to be much greater than the 10s of meters observed in this study (e.g., up to or beyond 1 km; Eiras et al., 2021). Furthermore, of the calls that were estimated to propagate beyond 2 km (i.e., calls with a received level above 150 dB re 1 µPa), 80% were produced within 10 m of the surface. In fact, only 25% of calls that were estimated to propagate beyond 1 km were produced at depth (below 10 m), compared to nearly half (45%) of all other calls.

As we do not currently have separate transmission loss equations that account for propagation differences arising from variations in depth, only one propagation model was applied to the call dataset. This meant that the predicted active space of some calls was well beyond 2 km. However, calls propagate more poorly in the upper water column due to interference caused by the sounds’ reflection off the surface (i.e., the Lloyd mirror effect, Richardson et al., 1995). Nearly 60% of all adult female–calf calls were produced within 10 m of the surface, with levels approximately 8 dB greater than those of calls produced at depth. If depth could be taken into account, it is likely that the estimated active space of these calls would be considerably reduced. As adult female–calf pairs tend to maintain a distance of 2.5 km from nearby conspecific groups, it appears they may use shallow calling as a strategy to reduce their active space and prevent unwanted receivers from detecting their calls while maintaining acoustic contact at “normal” levels (Indeck et al., 2021).

It is also important to note that active space here was calculated using received levels of *all* adult female–calf calls. While received levels of calls presumed to be from the adult female could be considered a proxy for source level (Parks et al., 2019; Videsen et al., 2017), received levels of many presumed calf calls were likely lower than their actual source levels (because of adult female–calf separation distance and call transmission loss). Therefore, although we potentially *overestimated* the active space of these pairs by not accounting for the shallow depth at which many calls were produced (i.e., accounting for the Lloyd mirror effect), we also potentially *underestimated* this space by including the received levels of calf calls. Additionally, although calls are believed to be detectable by conspecifics down to an SNR of 0, the level required for functional discrimination and effective communication is yet unknown. As such, further work should focus on estimating the transmission loss of calls according to the depth at which they are produced, measuring source levels of calls for both adult females and their calves, and determining the SNR threshold for call discrimination.

As resting pairs spend a greater proportion of time near the surface with little change in location (Table 1), their behavior and associated vocal activity may be affected more significantly by passing vessels than would be the case in other behavioral states. When resting, whales have been found to react more intensely to disturbances than during social or travel activities (Cantor et al., 2010). Specifically, adult female–calf pairs have been shown to take longer to return to predisturbance behavior than groups of other compositions (Williamson et al., 2016), as well as to exhibit an immediate and extended acoustic response (elevated call rate) when exposed to vessel noise (Parks et al., 2016). Although Fournet et al. (2018) also found that humpback whales increased the source levels of their calls as compensatory behavior for moderate levels of vessel noise generated 3 to 10 km away (i.e., a Lombard response), the results of Dunlop (2016) did not show comparable changes when vessels were much closer. As adult female–calf pairs produce low‐level calls with a limited communication range, this means that their vocal activity is especially susceptible to acoustic masking, particularly if they do not exhibit a Lombard response. Any resulting disruptions to communication exchanges between adult females and their calves could result in an increased risk of prolonged or permanent separation (Eiras et al., 2021).

In summary, this study demonstrates the adult female–calf humpback whale strategy for communicating during periods of increased separation risk while simultaneously maintaining acoustic crypsis to minimize unwanted detection. Adult females were separated from their calves more often when resting because of prolonged periods of time spent at depth. As the calls of these pairs have a consistently limited detection distance, shown to be primarily a conspecific avoidance strategy (Indeck et al., 2021), they compensate for this by increasing their rate of calling to ensure continued acoustic contact during these periods. Additionally, calls with higher received levels were more frequently produced when the adult female was less than 10 m from the surface, meaning that they are unlikely to propagate as far as those produced at depth. As such, the vulnerability of adult female–calf pairs to behavioral and acoustic disruption (e.g., energetic costs and inability to maintain contact), particularly during periods of rest, has potential implications for calf survival rates and represents a crucial approach for future noise studies.

## CONFLICT OF INTEREST

There were no actual or perceived conflicts of interest.

## AUTHOR CONTRIBUTION

**Katherine Laura Indeck:** Conceptualization (lead); Data curation (lead); Formal analysis (lead); Investigation (supporting); Methodology (equal); Visualization (lead); Writing – original draft (lead); Writing – review & editing (lead). **Michael Noad:** Conceptualization (supporting); Formal analysis (supporting); Funding acquisition (equal); Investigation (lead); Methodology (supporting); Resources (equal); Supervision (supporting); Visualization (supporting); Writing – review & editing (supporting). **Rebecca Dunlop:** Conceptualization (supporting); Formal analysis (supporting); Funding acquisition (equal); Investigation (lead); Methodology (equal); Resources (equal); Supervision (lead); Visualization (supporting); Writing – review & editing (supporting).

### OPEN RESEARCH BADGES

This article has earned an Open Data Badge for making publicly available the digitally‐shareable data necessary to reproduce the reported results. The data is available at https://doi.org/10.5061/dryad.98sf7m0jd.

## Supporting information

Appendix S1

## Data Availability

The data supporting this article are available through DRYAD (DOI: https://doi.org/10.5061/dryad.98sf7m0jd).
