# Supplementary material for: Humpback whale adult females and calves balance acoustic contact with vocal crypsis during periods of increased separation
Source: Ecol Evol. 2022 Feb 9;12(2):e8604. doi: 10.1002/ece3.8604 (PMC8826064; doi:10.1002/ece3.8604)
Supplement: Supplementary file 1 — Appendix S1 [file ECE3-12-e8604-s001.docx]

| **Table A1** Values of *a* and *b* used to estimate TL, for the octave bands and distances shown. Adapted from [Dunlop *et al.* 2013](file:///S:\Bookingin_Production\25-01-2022\ECE3\1\doc\ms.docx#_ENREF_14). | | | |
| --- | --- | --- | --- |
| **Octave centre frequency (Hz)** | **Distance (m)** | ***a*** | ***b*** |
| **63** | All distances | -5.0 | 20.4 |
| **125** | ≤ 580 | 3.2 | 16.7 |
|  | ≥ 580 | -16.14 | 23.7 |
| **250** | ≤ 890 | -4.3 | 18.3 |
|  | ≥ 890 | -24.3 | 25.1 |
| **500** | ≤ 890 | 3.7 | 14.6 |
|  | ≥ 890 | -32.4 | 26.8 |
| **1000** | ≤ 1100 | 2.0 | 15.0 |
|  | ≥ 1100 | -44.6 | 30.3 |
| **2000** | ≤ 1700 | -7.6 | 19.7 |
|  | ≥ 1700 | -61.2 | 36.2 |
|  |  |  |  |

**Appendix**
